# Surgical treatment of a distal radius and ipsilateral metacarpal hemophilic pseudotumor without recurrence or functional deficit: a case report

**DOI:** 10.3389/fped.2023.1053368

**Published:** 2023-05-30

**Authors:** Yingjie Wang, Zehui Lv, Xiying Dong, Bin Feng, Xisheng Weng

**Affiliations:** ^1^ Department of Orthopedic Surgery, State Key Laboratory of Complex Severe and Rare Diseases, Peking Union Medical College Hospital, Peking Union Medical College & Chinese Academy of Medical Sciences, Beijing, China; ^2^ Peking Union Medical College Hospital, Peking Union Medical College & Chinese Academy of Medical Sciences, Beijing, China

**Keywords:** intraosseous hemophilic pseudotumor, curettage and bone grafting, continuous follow-up, complete recovery, hemophilia A

## Abstract

**Background:**

Distal hemophilic pseudotumor (HP) occurring distal to the wrist appear to be secondary to intraosseous hemorrhage, which develops rapidly and should be treated primarily with long-term replacement therapy and cast immobilization. Surgical removal or even amputation is indicated when conservative management fails to prevent progression. Here, a practical strategy was described for those patients who cannot afford the cost of routine coagulation factor replacement therapy, namely immediate surgical curettage and bone grafting as well as continuous follow-up.

**Case description:**

A 7-year-old boy with a history of mild hemophilia A was admitted to our medical center because of a 2-year history of progressive swelling and pain around right forearm and hand. Coagulation factor VIII level was 11.1% of normal with no inhibitor. Radiographs revealed expansile swelling, bone destruction, and deformity of the distal right radius and the second metacarpal bone. He was diagnosed with distal HP. Surgical procedure of curettage and bone grafting was performed. The function and appearance of the right wrist were almost normal without discomfort at the 101-month follow-up. Significantly, the same patient was hospitalized again because of a year-long progressive swelling and pain around the left hand when he was 14 years old. X-ray showed multiple bone destruction of the left proximal phalanges of left thumb, middle finger and little finger with local pathological fractures. Surgical procedure of HPs including curettage and bone grafting was performed. Postoperative recovery was good, and the last clinical follow-up at 18 months after the operation displayed a satisfactory shape and functional outcomes.

**Conclusions:**

Curettage and bone grafting prove to be safe and feasible for patients with distal HP and continuous follow-up of patients with distal HP is very vital for timely finding and then treating successive HP in developing countries.

## Introduction

Hemophilic pseudotumor (HP) is a very rare but serious complication, which can lead to disability and even death of hemophiliacs (1). Its prevalence is 1%–2% in severe hemophiliacs with factor deficiency and can be up to 10% in patients with factor VIII or IX inhibitor (2), although it can also occur in moderate hemophiliacs (3). The pathology of HP was first described by Starker in 1918, which is basically an encapsulated hematoma with a thick fibrous capsule with calcification and ossification within (4). Over 90% of bleeding episodes in hemophiliacs occur within the musculoskeletal system (4). There are two classification systems based on anatomical locations for HP. HPs were divided into the proximal type and the distal type by Gilbert (5). The other classification system consists of the soft tissue type, the subperiosteal type, and the intraosseous type (6). The distal HPs, mainly occurring distal to the wrist and ankle, develop rapidly and seem to be secondary to intraosseous hemorrhage. Additionally, the distal HPs were primarily reported in children and adolescents (4). The most serious sequelae of HPs include pathological fractures, neurovascular compression, and uncontrollable bleeding (7, 8). Most reported cases highlighted the importance of early recognition and diagnosis of pseudotumor (5, 9, 10).

The reported lesions associated with musculoskeletal system included tibia, femur, hip, iliac, iliopsoas, and so on (11). However, there has not been successive intraosseous HPs in different locations till now. This study reported that a 7-year-old boy received surgical treatment of intraosseous HPs and bony allograft of the right distal radius and the second metacarpal bone for the first operation. The same patient received the second surgical removal of intraosseous HPs and bone allograft of the left first, third, and fifth proximal phalanges 7 years after the first operation. This patient had been followed up for 101 months since his first surgery at our medical center. For all we know, we reported the longest follow-up of a patient with hemophilic pseudotumor.

## Case description

**Figure 1:**
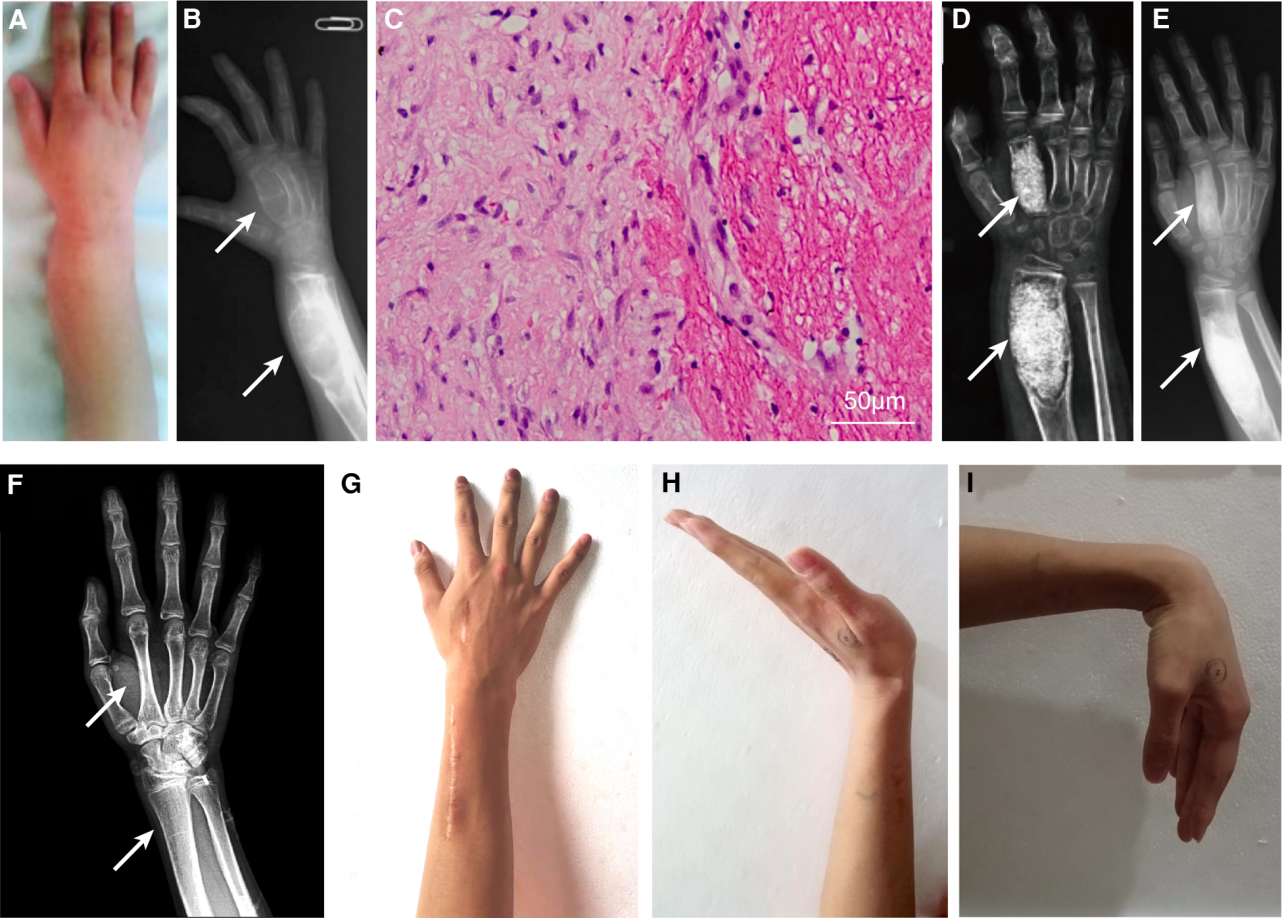
Clinical and radiographical results before and after surgery of the right distal radius and the second metacarpal bone, and pathological result. The swollen right distal radius on the dorsum lateral aspect (**A**). The preoperative x-ray (**B**); hematoxylin-eosin staining of pathological tissue showing fibrous tissue with chronic inflammation and blood clot (**C**); x-ray 2 weeks postoperatively (**D**); 5 months postoperatively (**E**); and complete recovery of anatomic structure at 101 months postoperatively (**F**). Appearance (**F**) and range of motion (**G–I**) of the right wrist at the latest follow up. White arrow indicates the locations of intraosseous hemophilic pseudotumor.

A 7-year-old boy with a history of mild hemophilia A was admitted to our medical center because of a 2-year history of progressive swelling and pain around right forearm and hand. His parents denied family history, any history of trauma or surgery. The patient had been diagnosed with fibrous dysplasia of bone and bone cyst, but did not receive any medical treatment before admitted to our medical center. Physical examination demonstrated that the right distal radius was swollen (50 mm × 30 mm × 20 mm) with well-defined margins and smooth surface on the dorsum lateral aspect (Figure 1A), with tenderness to palpation, and no significant abnormality was observed on the epidermal surface. The range of motion (ROM) of the elbow was normal, while pronation and supination of the right forearm and ROM of the right wrist were restricted.

Laboratory tests showed that coagulation factor VIII level was 11.1% of normal with no factor VIII inhibitor, and APTT time was 48.4 s (reference 23.3 s–32.5 s). Other indicators were mostly within normal reference ranges. Radiographs revealed expansile swelling, bone destruction, and deformity of the distal right radius and the second metacarpal bone. The x-ray presented with soap like change in the thin cortical bone and low density of medullary cavity (Figure 1B). He was diagnosed with distal HPs.

Considering the increasing pain and progressive bone cortex damage in the region of enlarging epiphyseal, surgical procedure of curettage and bone grafting was performed. The activity of factor VIII was maintained at 100% on the day of surgery by transfusion of recombinant factor VIII. Briefly, the lesions of distal radius and second metacarpal bone were curetted, and the bony defects were packed with allograft cancellous bone chips without internal fixation after a cortical window was made.

Recombinant factor VIII was intravenously transfused to reach the postoperative requirement: 50%–100% in POD (postoperative days) 1–3, 40%–80% in POD 4–6, and 30%–60% in POD 7–14. The pathology confirmed the diagnosis of intraosseous HPs with a predominance of blood clot, scattered fibrous tissue, and mild chronic inflammation (Figure 1C). The patient's forearm was immobilized by a splint for 4 weeks postoperatively, during which gentle wrist motion exercises were prescribed. Routine x-ray follow-up was scheduled at 2 weeks (Figure 1D), 5 months (Figure 1E), and 101 months (Figure 1F) postoperatively. X-ray at 2 weeks revealed successful implantation of allograft bone and satisfactory recovery at 5 months and complete recovery at 101 months postoperatively. Routine clinical follow-up indicated improved ROM and pain relief of the right wrist at 5 months, and the ROM and appearance of the right wrist were almost normal without discomfort at the 101-month follow-up (Figures 1G–I).

**Figure 2:**
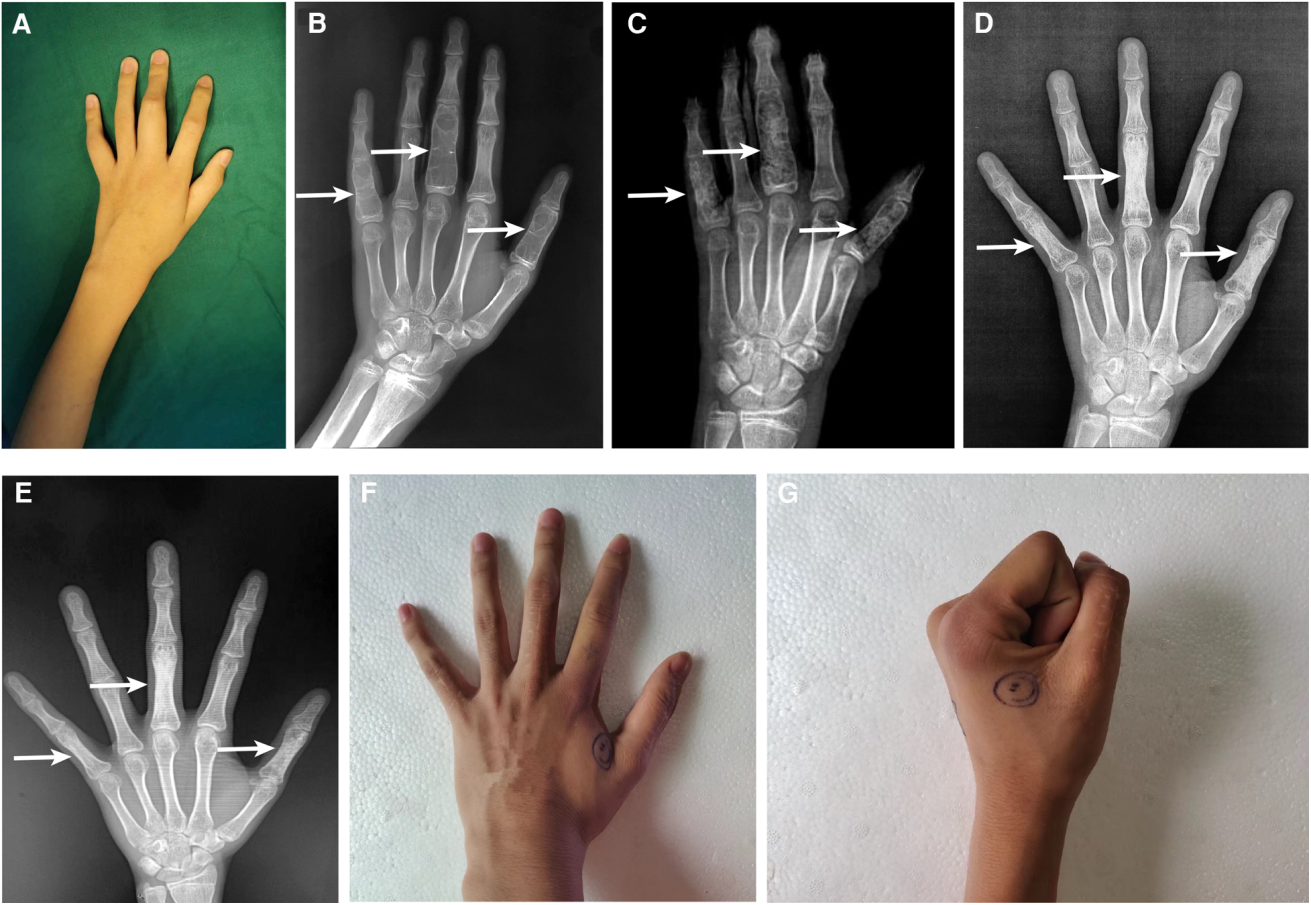
Clinical and radiographical results before and after surgery on the proximal phalangeal bone of left thumb, middle finger, and little finger. The fusiform eminence could be seen at the proximal phalangeal bone preoperatively (**A**). The preoperative x-ray (**B**); 2 days postoperatively (**C**); 7 months postoperatively (**D**); and 18 months postoperatively (**E**); white arrow indicates the locations of intraosseous hemophilic pseudotumor. The appearance of proximal phalanges (**F**) and the range of motion of metacarpophalangeal joints (**G**).

Unfortunately, the same patient was hospitalized for the second time because of 1 year-long progressive swelling and pain around the left hand when he was 14 years old. He denied any history of trauma. Physical examination showed that fusiform eminence could be seen at the proximal phalangeal bone of left thumb, middle finger (30 mm × 20 mm × 20 mm), and little finger (Figure 2A). Factor VIII level was 60.8%, and no factor VIII inhibitor was detected preoperatively. X-ray showed multiple bone destruction and thin cortical bones of the left proximal phalanges with local pathological fractures (Figure 2B).

The successive HPs, severely impairing the ROM of joints, were surgically resected on the account of the disappointing outcome and economic burden of coagulation factor replacement therapy alone. Surgical curettage of HPs in proximal phalangeal of left thumb, middle finger and little finger and bone allograft was performed under general anesthesia. The perioperative factor VIII replacement therapy was the same as the surgery performed 7 years ago.

X-ray on POD 2 showed a satisfactory bone graft (Figure 2C). Radiographical follow-ups was scheduled at 7 months (Figure 2D) and 18 months (Figure 2E) postoperatively, the results of which demonstrated a complete recovery of the shape and structure of proximal phalanges. Additionally, the last clinical follow-up at 18 months after the operation displayed a satisfactory shape (Figure 2F) and functional outcomes (Figure 2G). Following discharge, the patient complained of intermittent bleeding in the hips and iliopsoas after minor trauma, and 20 U/kg of factor VIII was given subsequently. The average daily concentration of coagulation factor VIII was about 4.0%. We will continue to pay attention to the treatment outcome of intraosseous HPs and the progress of hemophilia A by regular follow-ups.

## Discussion

Distal HPs were mainly caused by spontaneous bleeding in immature small bones of the hands and feet, which occurs mostly in young hemophiliacs with open epiphyseal growth plates (10). The acknowledged treatments of hemophilic pseudotumor include surgical removal, embolization, radiotherapy, puncture drainage, conservative therapy, fibrin glue or bone graft, hydroxyapatite filling, etc. (2, 12, 13). However, largely confined to case reports or small case series, there is no consensus for hemophilic pseudotumor treatment. Although there is no agreement on the first-line therapy for HPs, many studies suggested that surgical treatment had the best prognosis (14, 15). Coagulation factor replacement therapy combined with immobilization can be initially used to treat distal HPs (15). If the treatment is delayed, serious complications such as skin or abdominal fistula, bleeding, infection, sepsis, pathological fractures, and even death may occur (10). Untreated proximal HPs might eventually destroy soft tissues, erode bones, and produce neurovascular complications (10). Proximal HPs mainly occurring in the long bones (especially the femur) and pelvis of adult patients with hemophilia is usually refractory to conservative treatment. In general, surgical removal of the entire mass is reliable. Because it is prone to relapse, the wall and content of HP should be completely removed (16, 17). Nonetheless, surgical resection faces the risks for complications such as bleeding, recurrence of pseudotumor, and infection (13, 18). Surgical resection with coagulation factor replacement was safe and effective in most cases, which is consistent with our published study (2).

Continuous follow-up of patients with hemophilic pseudotumor is very significant. The incidences of pseudotumor in hemophilia patients in China (14) and Spain (19) are 1.12% (14/1,248) and 1.56% (21/2,280), respectively. Additionally, hemophiliacs in China (14) and Spain (19) developed pseudotumor at average ages of 40.0 and 23.4, respectively. The above data showed that the daily injection of coagulation factors seemingly did not reduce the incidence of pseudotumor, and advancing age was an independent risk factor for pseudotumor development due to two possible reasons: (i) the long lapse between the initial injury and pseudotumor development, (ii) the collective episodes of intramuscular bleeds from aging blood vessel.

Currently, the diagnosis of HP mainly depends on the patient's medical history and postoperative pathological results, it was recommended that a pathological diagnosis can be made through biopsy after replacement therapy with a small amount of coagulation factors before surgery and bone scans can be performed to rule out other tumors. It was noting that gene diagnosis could be employed for fetus. Concerning the treatment of HP, bone grafting can be promoted locally by using growth factors such as BMP-2 to facilitate integration between the graft and host bone after excision of HP. In addition, biologically active bone graft materials can be used to promote host bone ingrowth during local filling and reconstruction, such as rhBMP7 products including Medtronic's Infuse®, Stryker's OP-1 Implant®, OP-1 Putty®, and Osigraft®. While gene therapy might be the ultimate treatment for hemophilia patients in the future.

In conclusion, we reported the longest follow-up of a patient with hemophilic pseudotumor and the successive intraosseous HPs in different locations for the first time. Additionally, we provided a practical strategy that continuous follow-up combined with curettage & bone grafting prove to be safe and feasible for patient with distal HP who cannot afford the cost of routine coagulation factor replacement therapy in developing countries. More and larger sample size studies will be needed to further evaluate the long-term functional outcome of our strategies.

## Patient perspective

When I was 5 years old, my right forearm and right hand presented with swelling, pain, and limited activity, which seriously affected my study and daily life. It was misdiagnosed as fibrous dysplasia of bone, bone cyst, etc. initially, and was diagnosed as hemophilic pseudotumor at Peking Union Medical College Hospital when I was 7 years old. Me and my whole family were shocked. I feel that hemophilic pseudotumor will progressively lower the quality of life, because my family can struggle to pay the bills of daily factor replacement therapy. To our surprise, the function of my right hand was completely recovery after the operation, and no signs could be seen on the x-ray. Although I underwent surgery again because of the pseudotumor of my left hand when I was 14 years old, the function of my left hand is also completely normal. The key of maintain quality of life is that I have kept in contact with the hospital to facilitate timely communication of my condition and relevant treatment, which is very important for patients who want to maintain their quality of life but cannot afford the cost of daily factor replacement therapy.

## Data Availability

The original contributions presented in the study are included in the article, further inquiries can be directed to the corresponding authors.
